# Adaptable, wearable, and stretchable coils: A review

**DOI:** 10.1002/mrm.30428

**Published:** 2025-02-04

**Authors:** Thejas Vishnu Ramesh, Folk W. Narongrit, Joseph V. Rispoli

**Affiliations:** ^1^ Weldon School of Biomedical Engineering Purdue University West Lafayette Indiana USA; ^2^ Elmore Family School of Electrical and Computer Engineering Purdue University West Lafayette Indiana USA; ^3^ Department of Radiology and Medical Imaging University of Virginia Charlottesville Virginia USA

**Keywords:** adaptable, automatic tuning, RF coils, stretchable, wearable, wireless MRI

## Abstract

Over the last four decades, there have been various evolutions in the design and development of coils, from volume coils to the recent introduction of wireless receive arrays. A recent aim has been to develop coils that can closely conform to the anatomy of interest to increase the acquired signal. This goal has given rise to designs ranging from adaptable transmit coils to on‐body stretchable receive arrays made using fabric or elastomer substrates. This review covers the design, fabrication details, experimental setup, and MRI results of adaptable, wearable, and stretchable MRI coils. The active and passive automatic tuning and matching strategies are examined with respect to mitigating signal‐to‐noise ratio reduction when the coil form is altered. A brief discussion of wireless MRI coils, which provide a solution to overcome the cabling issues associated with MRI coil development, is also included. The adaptable, wearable, and stretchable coils and various coil tuning techniques represent innovative radiofrequency coil solutions that pave the way for next‐generation MRI hardware development.

## 1 INTRODUCTION

Radiofrequency (RF) coils are the magnetic field antennas through which images are acquired in MRI. To minimize resistive losses, surface receive coils traditionally were formed from copper wire and evolved to include printed circuit board (PCB) technology.^1^, ^2^, ^3^, ^4^, ^5^, ^6^, ^7^, ^8^, ^9^, ^10^ The high sensitivity achievable by rigid coils can nonetheless obtain limited signal‐to‐noise ratio (SNR) because of the inflexibility of the coil when placed over anatomical curvatures. The introduction of flexible copper‐clad laminates allowed thin PCB coils to bend around the region of interest (ROI).^11^, ^12^, ^13^, ^14^, ^15^, ^16^, ^17^, ^18^, ^19^, ^20^ Closer proximity to the ROI increases the sample noise dominance of the coil.^21^, ^22^ Flexible PCB coils still could lack conformity around the ROI, leading to a reduced filling factor and SNR and spurring new design approaches.^23^ Adaptable coils, which house flexible PCB arrays on adjustable coil housing, were developed to overcome the lack of conformity of a flexible coil to obtain maximum filling factor across various subjects. The adaptable coils served as a precursor to wearable coils developed using coaxial cables, thermoplastic polymers, or metal clad fibers embedded in garments. Wearable coils do not need rigid housing to be near the anatomy of interest, thus taking advantage of the close fit to increase the SNR compared to adaptable coils. Stretchable coils are a subset of wearable coils that incorporate elastane substrates to elongate a wearable coil for use over a wide range of the population. The objective of this paper is to review existing literature regarding the development workflows, advantages, and limitations of the techniques used to fabricate adaptable, wearable, and stretchable coils. The literature was surveyed using the PubMed database with the following search terms: MRI, coils, stretchable, wearable, adaptable, and wireless. The paper incorporates the following:

- A review of adaptable coils, including a subsection on coils that require adjustable mechanical housing to encompass the ROI, and a second subsection on modular array development.
- A review of wearable coils developed on fabric and nonfabric substrates.
- A review of stretchable coils developed on fabric and non‐fabric substrates.
- A brief review of automatic coil tuning and matching methods used to mitigate SNR loss in wearable and stretchable coils.
- Figures depicting coil development techniques that are mechanically complex or involve unique fabrication and coil placement.
- A discussion of the various coil development methods, RF coil cabling considerations, the role of wireless coils, and the future direction of MRI coils.
- A literature summary table highlighting the advantages and limitations of each technique.

## 2 ADAPTABLE COILS

Adaptable coils are adjustable arrays that encompass an anatomical region across a wide range of the population to provide consistent SNR. There are two types of adaptable coils: those featuring mechanical fixtures to readjust the coil form to fit different sized subjects, and those using modular design or novel material engineering techniques to rapidly develop custom‐fit coils.

### 2.1 Mechanically adaptable coils

Coils that can be adapted to varying subjects through mechanical adjustments use hinges, knobs connected to the mechanical housing, sliding levers, and bellows to achieve the intended dimensional variations. Care must be taken to maintain robust tuning, matching, and decoupling to achieve maximum SNR in all states of coil adjustment.^24^, ^25^, ^26^, ^27^, ^28^ In 2008, Adriany et al. developed a 16‐channel mechanically adjustable transverse electromagnetic resonator head coil.^29^ This coil used capacitor patches made of copper tape on a polytetrafluoroethylene substrate for interelement decoupling. Although adjustable for varying head sizes, the coil required frequent retuning and matching to maintain optimum B_1_^+^ homogeneity and transmit efficiency, which would present a challenge in clinical environment.

**FIGURE 1:**
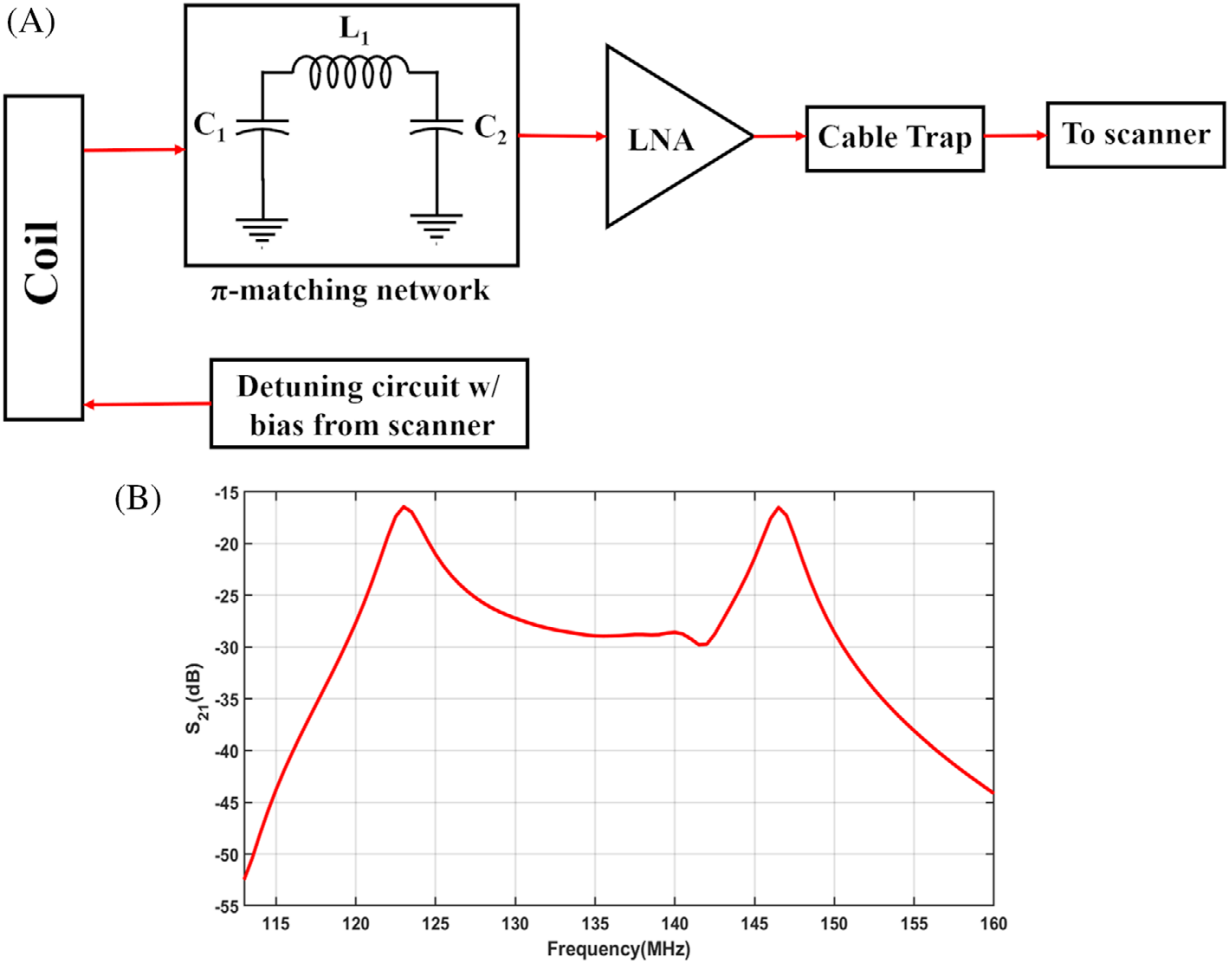
(A) Schematic of the receive coil system used in the adaptable eight‐channel wrist array. (B) The double hump resonance obtained by adjusting the π‐matching network in (A).

**FIGURE 2:**
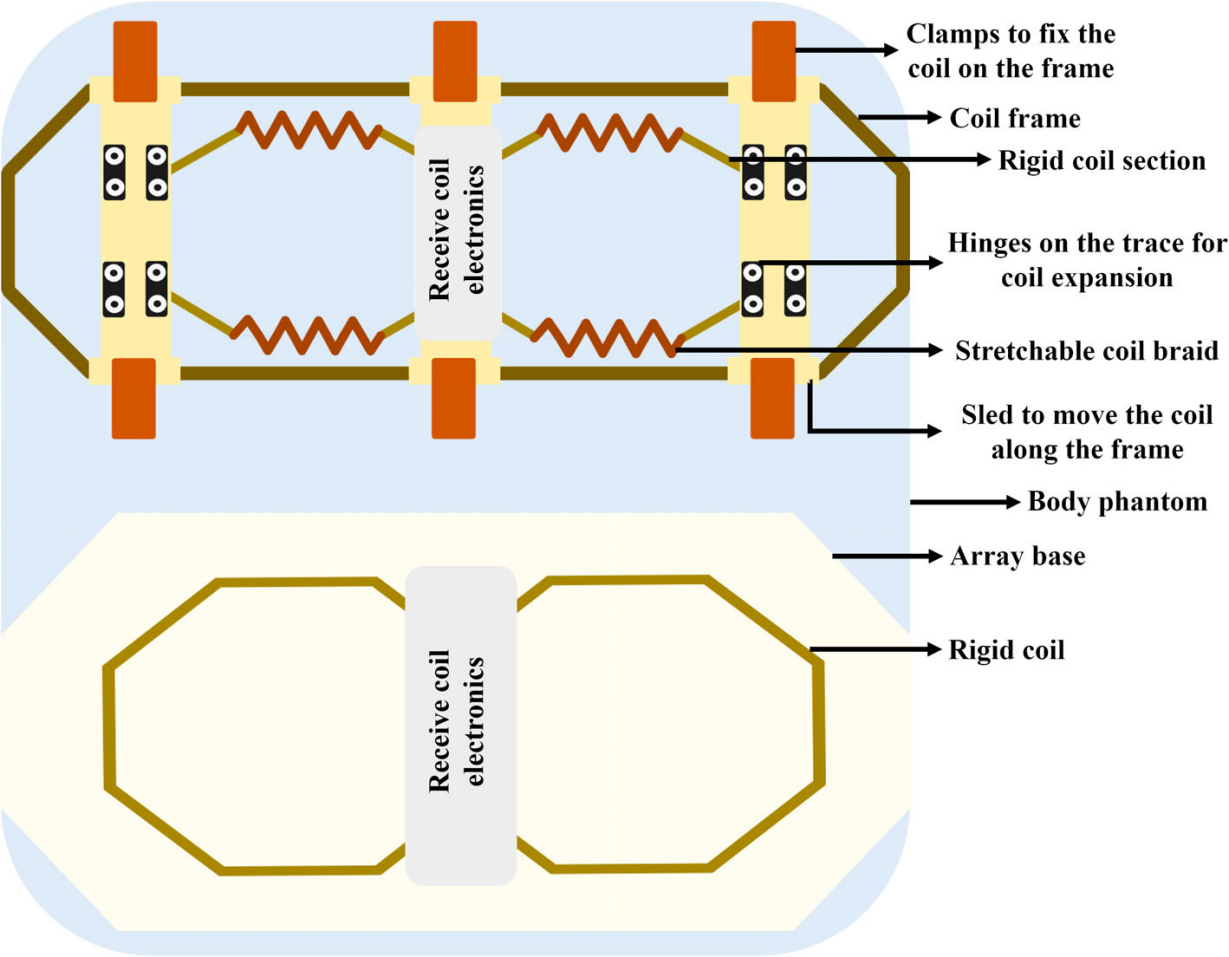
The longitudinally stretchable two‐channel receive array for knee imaging at 1.5 T (*top*) and the rigid copper‐based array for performance comparison (*bottom*), as demonstrated by Gruber et al.^31^ The arrays were placed on a body phantom using a frame for the stretchable array and a flat base for the rigid array. The geometric decoupling of the array beneath the receive electronics is not shown in the illustration.

When a coil is physically adjusted, the preamplifier decoupling network can be optimized instead of tuning and matching the coil to achieve a suitable SNR. In an eight‐channel wrist array, Nordmeyer‐Massner et al. used variable capacitors in the π‐matching network before the high‐input‐impedance preamplifier.^30^ The π‐matching network resonated at the same frequency as the coil, thus creating a double hump structure that incorporated the resonance frequency in the saddle, as shown in Figure 1. The reason for choosing the double hump frequency response is that the change in load does not affect the resonance frequency saddle, and changes to the coil coupling will also not change the frequency response, thus providing steady SNR values for different coil configurations. Gruber et al.^31^ designed an adjustable coil for knee imaging, combining rigid copper tape and stretchable conductive braids, as shown in Figure 2. This design allows for adjustment to fit various knee sizes. However, stretching the coil can lead to SNR loss due to variation in noise matching to the preamplifier, which can be overcome using auto‐tuning and wide‐band matching techniques.

**FIGURE 3:**
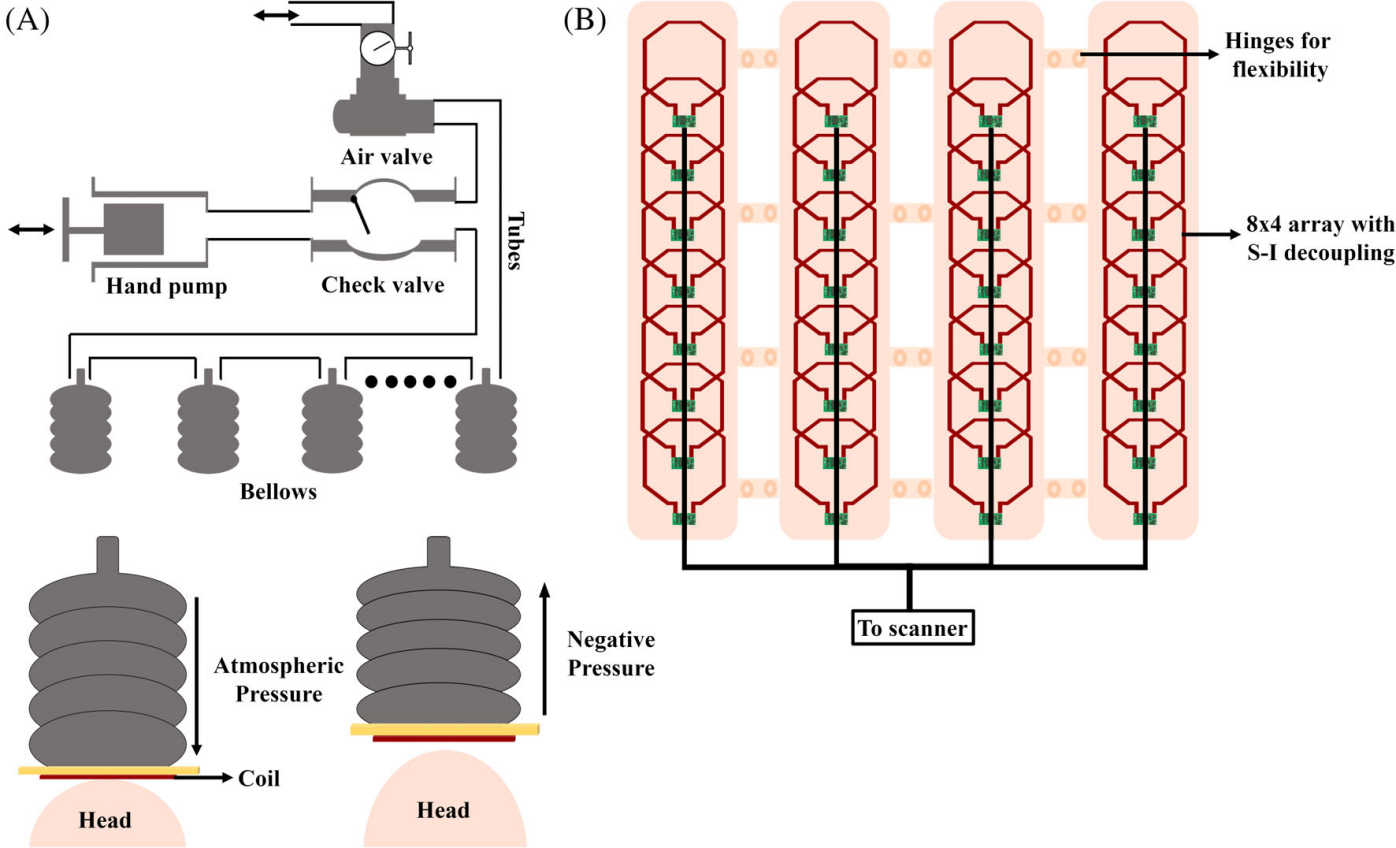
(A) The pressure control system as demonstrated by Lopez Rios et al.^43^ Negative pressure is created using the vacuum hand pump, which is maintained using a check valve, and atmospheric pressure is established using an air valve. The pump and valves are connected to the expandable bellows through a network of tubes. The negative pressure due to the vacuum pump compresses the bellows, resulting in coil movement away from the head, whereas the reverse displacement occurs when atmospheric pressure is restored. (B) The 32‐channel anterior array of the 64‐channel 3T pediatric body coil as described by Zhang et al.^44^ The rigid posterior array is not shown in the illustration. The hinges between the anterior array columns make the coil setup semiflexible over the abdomen.

The development of mechanically adjustable coils for adult subjects is complex, but the development of pediatric coils,^32^, ^33^, ^34^ especially adjustable coils, can present greater challenges. The coil must accommodate a wider range of patient sizes while, in the case of transmit coils, maintaining safe specific absorption rate (SAR) levels. For receive‐only arrays, the reduced signal availability due to the smaller body surface area in pediatric patients should be considered.^35^, ^36^, ^37^, ^38^, ^39^ The coils must also include a sufficient gap for intravenous cannulation of the child, if necessary, along with padding to limit motion in the event of an awake scan.^40^, ^41^, ^42^ A 13‐channel adaptable receive array was developed for brain imaging of neonates at 3 T, which can accommodate head sizes from 6 to 27 weeks.^43^ Each coil in the 13‐channel array was adjusted individually through a network of bellows embedded in the frame, using an air pump; the mechanism is depicted in Figure 3A. Individual motion of the coils combined with preamplifier decoupling provided over −30‐dB isolation between neighboring and next‐neighboring elements while exhibiting robust SNR across small, medium, and large head sizes.

Adult body coils have often been used for pediatric MRI, even though the coil is physically inefficient for imaging children, which causes issues such as limited parallel imaging performance and reduced image quality.^45^, ^46^, ^47^ A semiflexible 64‐channel array was developed to image the torso of children.^44^ The coil was divided into anterior and posterior sections consisting of 32 channels each. The 32‐channel anterior coil consisted of individual flexible PCB‐based coils in an 8 × 4 matrix, which were decoupled from the superior–inferior direction while maintaining a gap in the left–right direction, as shown in Figure 3B. The gaps were large enough to house hinges in between the columns of coils to make the anterior coil semiflexible, fitting snuggly around the children, thus minimizing SNR loss. A more economic and ergonomic design is needed compared with existing solutions, to realize the clinical utility of adaptable coils. A safety monitoring system can also be incorporated to monitor and report mechanical failure, specifically in coils for young children and neonates, with a penalty of increased coil cost and complexity.^42^ Auto–coil adjustment would improve over mechanical adjustments, and such an implementation should not increase scan times significantly, especially for pediatric populations in whom faster scan times are needed.^43^

### 2.2 Customized coils

Customized coils refer to coils designed for specific individual requirements or modular arrays that fit any part of the anatomy. The main motivation for modular arrays is to reduce the time and resources needed to develop anatomy‐specific coils and instead fabricate coils that can be rearranged to image any part of the body. Modular arrays also ensure direct access to leads, ventilation, and anesthetic tubes, which can be difficult to achieve using conventional coils due to the enclosures surrounding the coil. The first implementation of a modular array used eight single‐channel coils for cardiac imaging at 3 T.^48^ The coils were not geometrically decoupled but relied on preamplifier decoupling using π‐matching networks connected to high‐input‐impedance preamplifiers to minimize noise due to interelement coupling. Geometric overlap of coils was avoided in favor of non‐overlapped but equally spaced coils because of improved signal sensitivity and mechanical freedom.

**FIGURE 4:**
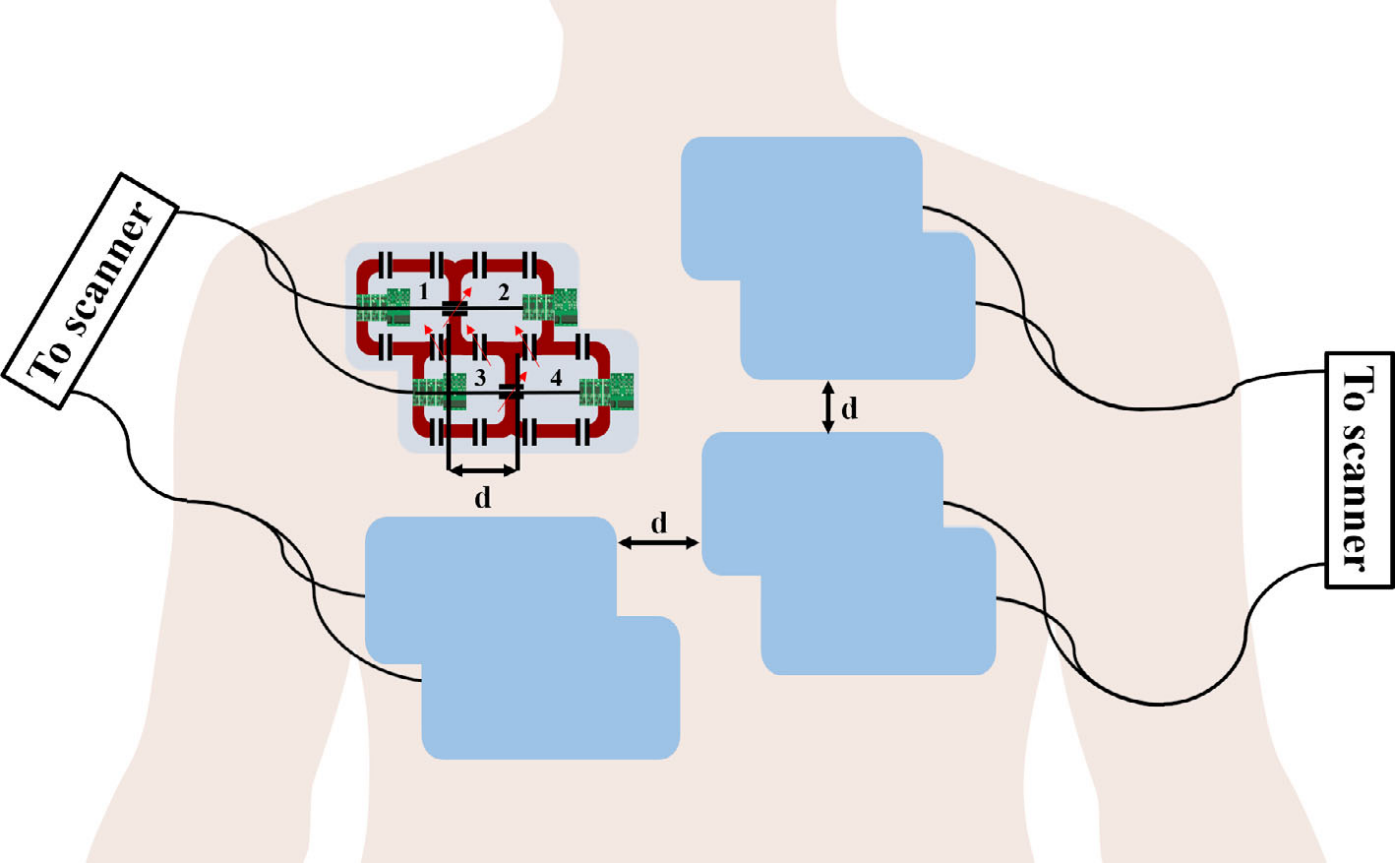
The modular 16‐channel anterior array as described in Graessl et al.^49^ The bottom row of each 2 × 2 array is shifted by approximately one diameter (d) to better decouple the coils at the edges (Elements 1 and 4). Adjacent elements share a trace with a variable capacitor (*red arrows*) for capacitive decoupling. Each 2 × 2 array is shifted by 3 cm for optimal intermodule decoupling. The 16‐channel flat posterior array is not shown.

To realize coil modularity with parallel imaging, a 32‐channel transceiver array was developed for two‐dimensional cardiac CINE imaging at 7 T.^49^ The 32‐channel coil was divided into eight groups of 2 × 2 arrays (four groups anterior, four groups posterior), as shown in Figure 4. The two rows in a four‐channel array were decoupled by shifting a row by approximately one diameter to minimize coupling between Elements 1 and 4. This type of array design is advantageous where the coil groups can be rearranged to cover any anatomy and ensures that no electrical adjustment is required before scanning. The only limitation is the inability to perform three‐dimensional (3D) CINE imaging due to limited spatial resolution, as dictated by clinical recommendations.^50^, ^51^ A flexible and modular seven‐channel array was demonstrated for brain imaging at 3 T.^52^ In practice, care must be taken to ensure that individual coil modularity is avoided in favor of modularity of subsets of coils for easier patient setup and better electrical and mechanical robustness. In 2023, high impedance coil (HIC) elements based on coaxial cable were used to form a modular array.^53^ Although a novel block‐by‐block construction approach, there was a SNR penalty when conformed around foot, ankle, and hip when compared with commercial coils, due to the inherent gaps between the array subsets when conformed around the complex curvatures.

**FIGURE 5:**
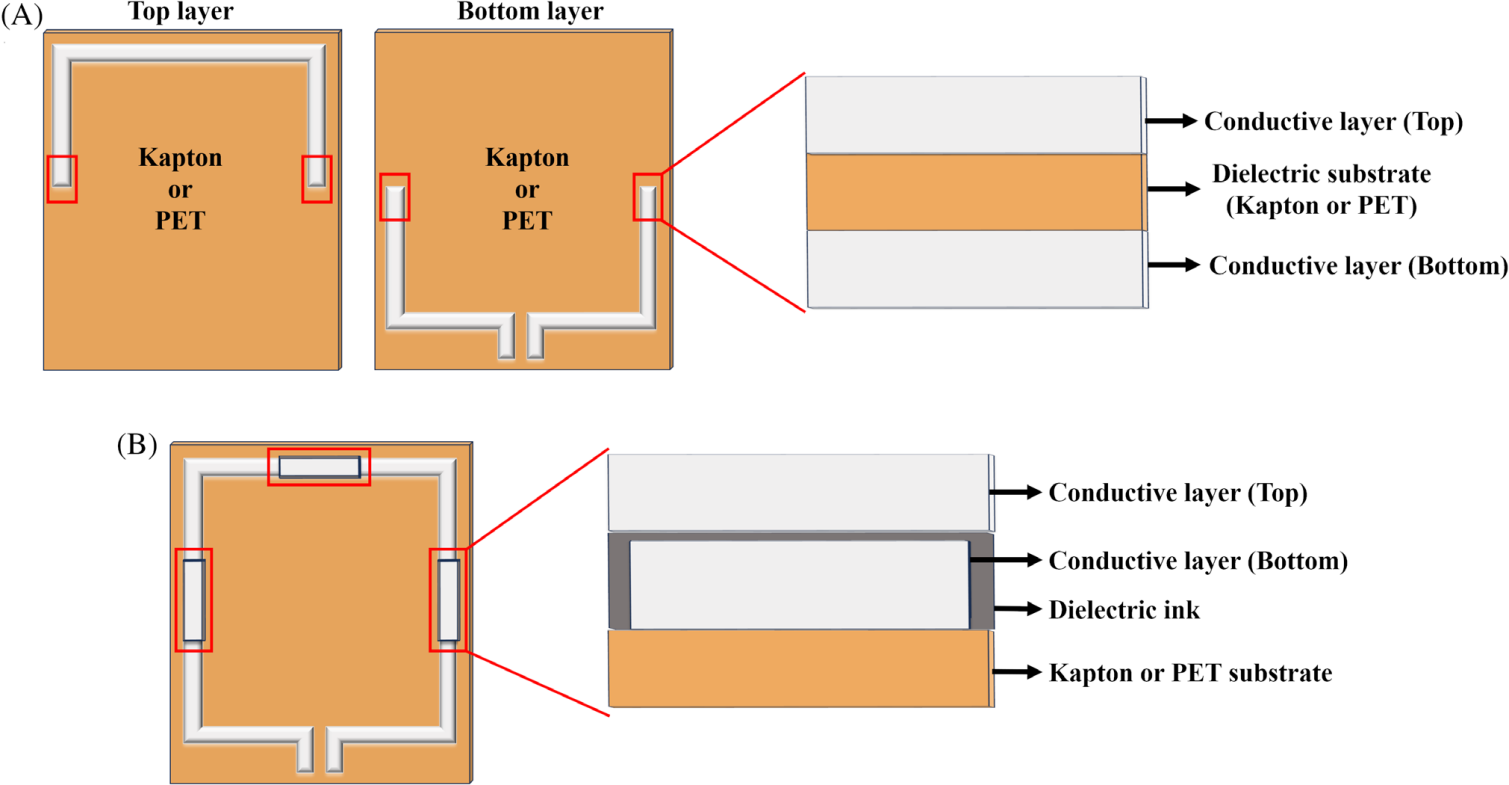
(A) Top layer of the conductive trace printed on one side of the flexible substrate, and the second half of the coil printed on the other side of the substrate. The composition of the dielectric substrate between two conductive layers is like a parallel plate capacitor. This method can be adopted for both inkjet printing and screen printing of MRI coils. (B) Alternate method for manufacturing tuning capacitors using the screen‐printing method. The complete coil loop is formed first by printing a conductive layer on top of a flexible substrate. The initial metal layer is covered with a dielectric resin on top of which another short trace of conductive layer is deposited to form the tuning capacitors.

Inkjet printing on flexible substrates is one of the techniques used to quickly prototype RF coils.^54^ In the inkjet printing method, silver suspension was ejected onto a Kapton base that served as both the substrate and dielectric material for the capacitors, as shown in Figure 5A. The limitation is that the printed capacitors cannot be adjusted for tuning of the coil to the Larmor frequency. Printing parameters such as speed, pixel (dot) spacing, and sintering time of the ink must be carefully evaluated to achieve the desired printing result. The nozzle size cannot be increased beyond a certain extent, as the kinetic energy of the ejected droplets produce an unstable print.^55^, ^56^, ^57^ These considerations and constraints make inkjet printing a complex process to manufacture MRI coils. The adaptive image receive (AIR) coil, which is an array made of lightweight, flexible link resonator structure that is proprietary to GE Healthcare, is another example of highly adaptable coils.^58^, ^59^, ^60^ The distributed capacitance design with lattice baluns used to transform impedance for preamplifier decoupling provides immunity between elements in an array. The AIR coil demonstrated the ability to be highly decoupled without any blocking circuits at ^129^Xe and ^3^He Larmor frequencies for multi–nuclear lung imaging.^61^ Recent iterations of the AIR coil have been used both for detecting the MR signal from the anatomy and for B_0_ shimming,^62^ which was inspired by similar rigid coil‐based designs.^63^

In recent years, two additional techniques have emerged to fabricate custom coils: spray coating and vacuum forming. Spray‐coated coils were used to image the human neck at 3 T.^64^ Spray coated coils offer simpler tuning process than inkjet‐printed coils but require scraping away the spray‐coated layers for coil tuning. Although the Q_unloaded_ of the spray coated coil was less than the copper foil coil, Q_unloaded_ > 100 will only increase the SNR by up to approximately 3%, as in this regime the patient loading affects the SNR more than Q_unloaded_.^65^, ^66^, ^67^ The vacuum‐formed coil technique was used to develop an eight‐channel array to image the visual cortex.^68^ Vacuum‐formed coils, although versatile and customizable, require significant time for plating and precise alignment between simulation and production models. The spray coating and vacuum forming techniques allow for capacitive, inductive, or geometric decoupling of array elements.

## 3 WEARABLE COILS

The goal of developing wearable arrays is to provide a close fitting, lightweight, custom RF coil solution to enhance image quality and patient comfort. Wearable coils also enable flexion studies of musculoskeletal anatomies, such as the knee and wrist, to improve postinjury or postoperative recovery. There are two main techniques for implementing a wearable coil: using conventional electronic materials paired with fabric and using fabric as the electrical substrate. In this section, the various methods used to implement wearable coils are discussed.

### 3.1 Wearable coils using non‐fabric materials

The earliest implementation of a cable as an MRI coil was the use of a flat cable for knee imaging based on monopole antenna theory.^69^, ^70^ A 60‐cm flat cable (one‐quarter wavelength at 3 T) with 60 individual parallel elements was tuned and matched to image the knee of a volunteer. The flat cable coil has the potential to be a wearable coil, especially for the knee, but imaging other anatomical regions may be difficult given the length of the coil. More research is needed to optimize the size of each cable element, and the distance between each element for imaging various anatomies.

Screen printing is a process in which a stencil design is transferred onto a substrate using ink and a squeegee through a mesh screen. The screen‐printing process was adopted for the development of flexible coils that can be incorporated into clothing for adult and pediatric patients.^71^, ^72^ There are two ways to develop MRI coils using a screen‐printing process. The first method is analogous to the inkjet printing method with the two halves of the coil on either side of the substrate, thus forming capacitors using the substrate as a dielectric. The second method printed the entire loop on one side of the substrate and covered the areas along the coil in which capacitors were to be formed with dielectric ink. Conductive ink was traced on top of the dielectric ink to form the tuning capacitors of the coil. The cross sections of both methods are shown in Figure 5. The neighboring elements in the four‐channel, screen‐printed spine array were geometrically overlapped for decoupling, wherein the neighboring elements were printed on alternate sides of the substrate. The conductor, substrate, and dielectric materials chosen must ensure that the coil is flexible and MRI‐transparent.^72^ The main limitation of the scalable screen‐printing technique is the careful control required to maintain both the appropriate dielectric ink composition and the area of the conductive ink to form the capacitor.

A trellis coil design, using layered crisscrossed nylon slats with copper elements, has been adopted for knee imaging at 3 T^73^ and body imaging at 7 T.^74^ This approach offers proximity to the ROI and minimizes decoupling changes due to positional shifts. The nylon substrate can be wrapped around cylindrical ROIs to increase SNR. Although the trellis structure effectively images cylindrical anatomical regions like the knee and thigh, the adaptability to more complex geometries remains to be explored.

Coaxial cables are used to develop transmit and receive coils owing to their intrinsic isolation, which aids decoupling between neighboring elements in an array.^75^, ^76^, ^77^ Current flows in the outer shield are the result of oscillating magnetic field during the receive mode. The discontinuities along the shield induce a voltage, which induces current flow in the inner side of the shield that is isolated from the outer shield by skin effect.^78^, ^79^ The receive coil was developed by interrupting the shield on the upper part of the coil and the core on the lower part of the coil. The resonant frequency is determined by the length of the cable, but the electrical length of the cable can be altered by adding additional gaps in the shield or multiple turns of the cable. For a 100‐mm‐diameter loop, conventional PCB‐based coil showed better decoupling at a 20‐mm overlap, whereas there was no predefined overlap for the coaxial cables due to very low coupling at all overlap distances. Interelement coupling of coaxial cable coils is primarily driven by electric‐field coupling to the load, and the cable shield somewhat prevents coupling between the inner shield and the sample.^77^, ^80^ A five‐channel coaxial cable‐based array was developed to image the carotids at 7 T.^81^ The concept of coaxial cable coils was extended to knee imaging at a low field strength of 0.55 T.^82^ This was achieved by creating a shield separation, while the core of the cable was left floating to improve coil efficiency. The improved coil efficiency comes at the cost of increased sensitivity to critical overlap for geometric decoupling than a purely coaxial coil.

HICs, which are derived from wireless power transfer systems,^83^, ^84^ use tailored coaxial cable transmission lines as the coil loops. The HIC is tuned by adjusting the cable length, relative permittivity of the dielectric, and outer shield and inner core diameters. Broadband detuning using HIC is achieved by shorting the gap between the inner core and outer conductor to eliminate surface currents across a broad spectrum using positive‐intrinsic‐negative (PIN) diodes to achieve the required level of detuning. In the HIC method, interelement decoupling is achieved by the very low‐input impedance at the preamplifier, as opposed to the high‐input impedances used in traditional low‐impedance coils. The HIC technique was first implemented to develop a 24‐channel glove array for imaging human hand and wrist at 3 T, in which the SNR of the HIC coil was shown to be higher than traditional PCB‐based coils.^85^ A limitation of the HIC setup is the reduction in coil size when the field strength increases. The change in field strength also necessitates different cable dimensions and dielectric substrates, which makes coil development expensive. The HIC concept cannot be applied for transceiver coils, as the decoupling scheme depends on the preamplifier. Despite the limitations, the HIC concept has been adopted for the development of a 28‐channel wearable coil to image the breast in both prone and supine positions,^86^ a 23‐channel array for imaging the human neck at 3 T,^87^ and an eight‐channel array for wrist imaging at 3 T.^88^

**FIGURE 6:**
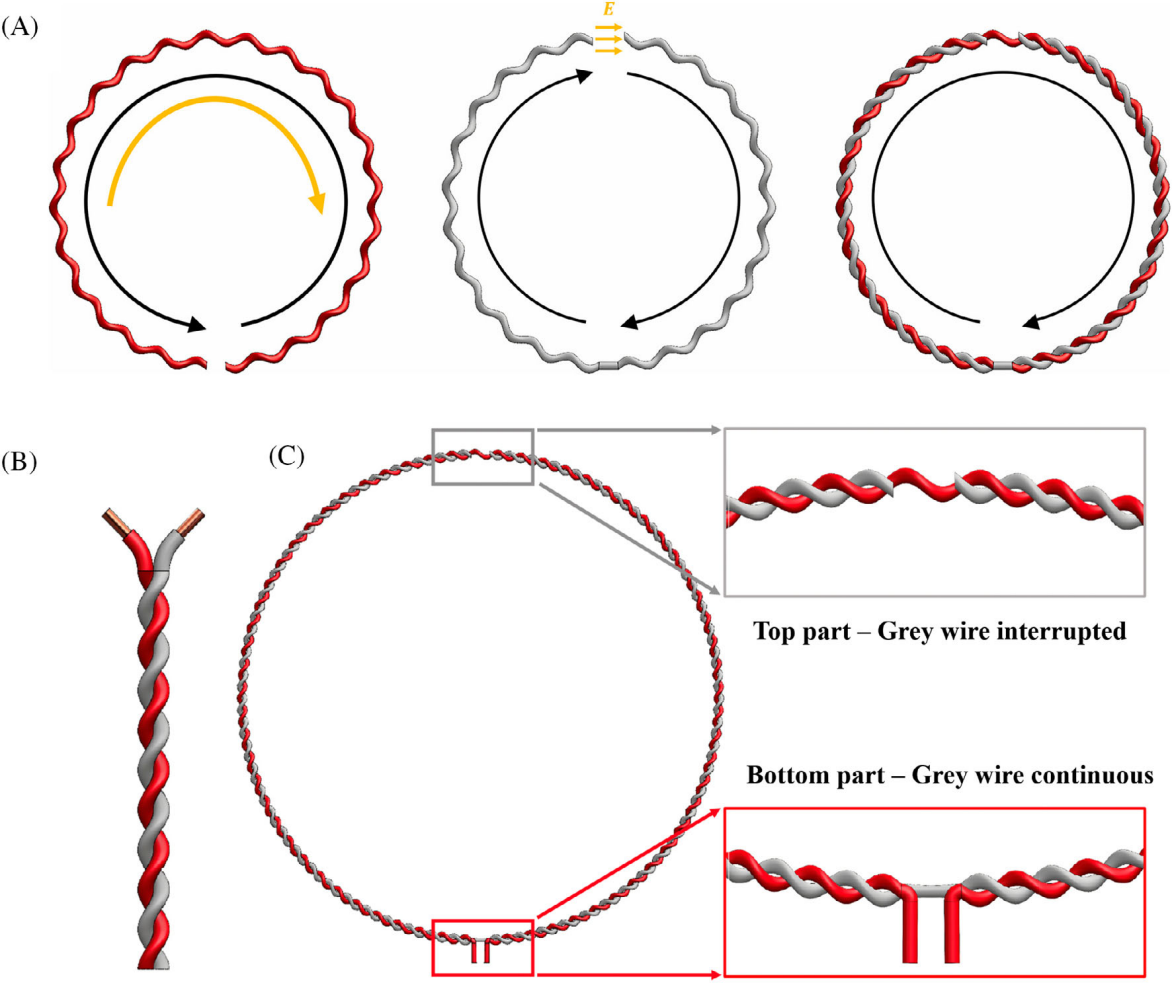
The twisted pair transceive coil demonstrated in Vliem et al.^92^ (A) When a counterclockwise current is excited on the red (signal) wire, a clockwise current is introduced in the gray (shield) wire. The high potential difference at the gap in the gray wire induces a current on the red wire flowing in the opposite direction as the original current. The induced current causes partial cancellation of the current in the red wire, thus making the gray wire the primary current carrier (clockwise current in the combined wires figure). (B) The twisted pair cable. (C) The fabrication of the twisted pair transceive coil. A gap is introduced on the gray (shield) wire at the top and a gap is introduced on the red (signal) wire in the bottom to act as the feed point. Images are adapted with permission from Vliem et al.^92^

Twisted‐pair cables are used to fabricate both transceive and receive‐only MRI coils.^89^, ^90^, ^91^, ^92^ In the case of receive‐only coils at 3 T, a predetermined length of twisted‐pair wire is cut followed by removing the insulation at three different points. The twisted pair is then joined to form a loop, with the required tuning and match reactance provided by the wires in the pair. The twisted‐pair coil provided SNR comparable with a traditional copper wire loop coil. The feed braid required additional shielding due to leakage of electric field into nearby lossy material when loaded. Common‐mode current traps can be used to reduce the coupling between the feed braids in a twisted‐pair receive‐only array. The decoupling, SAR, and transmit efficiency of the twisted pair coil were evaluated in a transceive array prototype. The twisted pair cable was formed into a 10‐cm‐diameter loop with the insulation removed at both ends of the loop, as shown in Figure 6. The amount of insulation removed influences the resonance frequency of the coil. The greater the number of twists, the better the coil return loss with low coil impedance and improved interelement decoupling in the array. An increase in cable twists reduced the B_1_^+^ efficiency and SAR of the transceive coil at 7 T. The robust decoupling combined with the absence of a rigid dielectric material makes the twisted pair cable insensitive to coil shape deformation when compared with coils developed with coaxial cables. The twisted pair transceive coil suffered from reduction in B_1_^+^ efficiency when moved away from the sample, as the coil primarily exhibits capacitive coupling to the sample.

**FIGURE 7:**
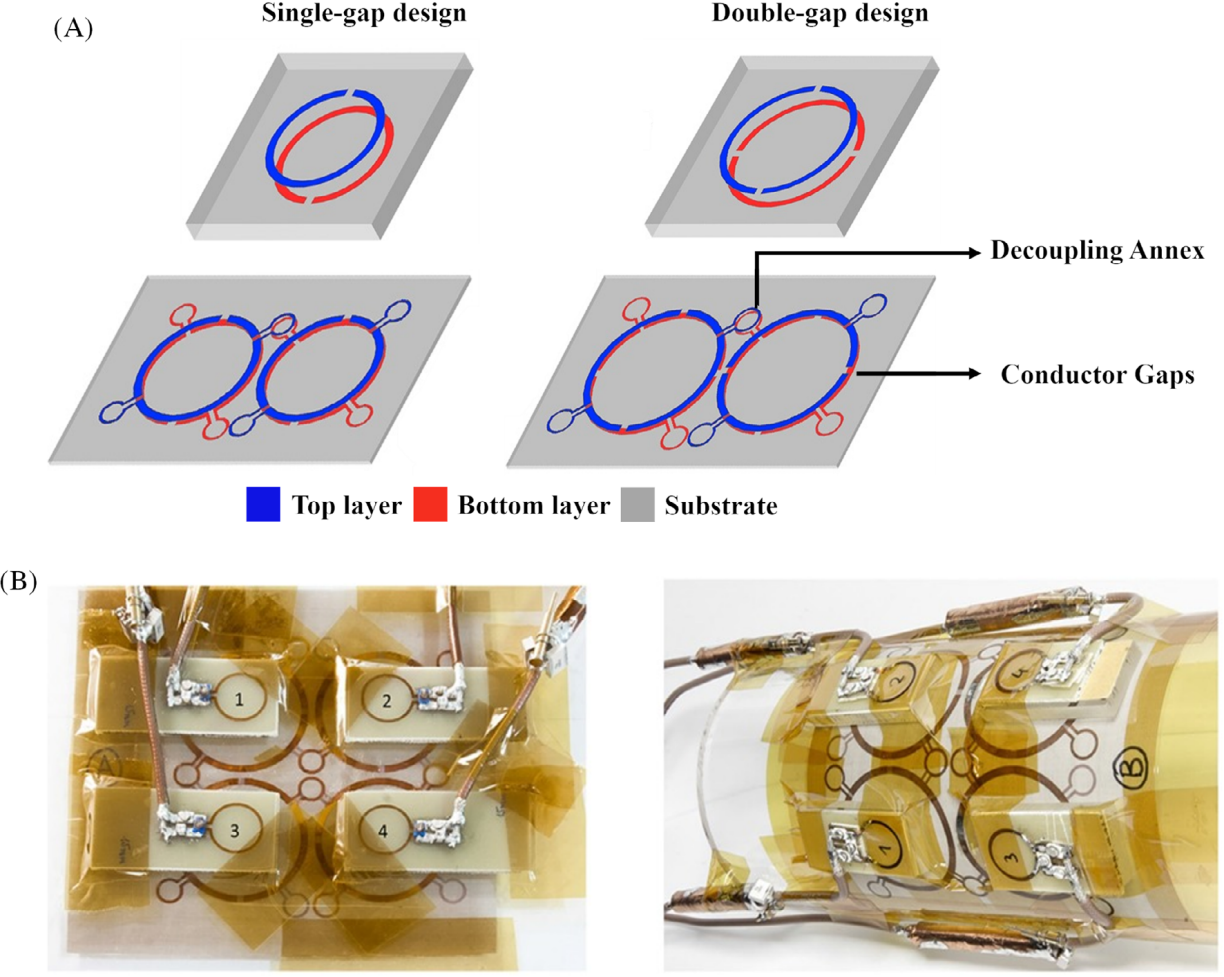
The transmission line resonator (TLR) coils shown in Kriegl et al.^94^ (A) The single‐gap (*left*) and dual‐gap (*right*) design of TLR coils. The single channel coils are shown at the top, and the two‐channel array configurations with decoupling annexes are shown at the bottom. (B) The four‐channel TLR array used on a flat phantom (*left*) and on a cylindrical phantom (*right*), which demonstrates the flexibility of the TLR array. Images are adapted with permission from Kriegl et al.^94^

The coaxial cable, twisted pair, and HIC coils are transmission line resonator (TLR)–based coils that require lumped elements. Parallel plate TLR coils do not require lumped elements on the coil and use gapped conductors with a dielectric substrate.^18^, ^93^, ^94^, ^95^ This creates a distributed component model, thus making TLR coils more flexible than flexible coils with lumped elements, as shown in Figure 7. Traditional decoupling techniques, such as precise geometric overlap and lumped components between coil elements,^96^, ^97^ are difficult to achieve with TLR coils. The fabrication of parallel TLR elements for geometric decoupling would require multiple layers when compared with a two‐layer PCB coil. Interelement decoupling is provided by creating coil annexes between the top and bottom resonators to cancel the mutual inductance. Rings can be created over each coil element for decoupling instead of annexes, which allow free placement of the TLR coils to improve sensitivity profiles. Capacitive decoupling can be achieved by connecting the capacitor networks at a specific position along the TLR, and inductive decoupling can be complicated in dense arrays. The main limitations of the parallel plate TLR design are the limited 3D deformation due to the parallel plate design and limited freedom in decoupling multiple TLR elements in an array. The twisted pair and parallel plate TLR coils showcase the ability to develop low‐profile MRI coils without lumped elements.

### 3.2 Wearable coils on fabric

The earliest implementation of an on‐fabric, receive‐only wearable coil was the six‐channel brachial plexus array developed using tinned copper braids on a fabric substrate.^98^ The design demonstrated the ability to fit small coil elements to image contorted, difficult‐to‐image anatomical structures like the brachial plexus.^99^ Wearable coils that use a fabric as a substrate often use conductive threads, which have been previously used in “smart” clothes and on‐body radiofrequency identification applications.^100^, ^101^, ^102^ The conductive thread is either stitched manually or fed into a sewing machine. In 2019, Zhang et al. provided a glimpse into the development of wearable coils using conductive threads by fabricating a two‐channel “electrotextile” array.^103^ The array was constructed using a silver and copper coated conductive thread consisting of 40 filaments (Liberator 40; Syscom Advanced Materials, Columbus, OH, USA) and exhibiting direct‐current (DC) resistance of 3 Ω/m. The tuning and matching capacitors were placed directly onto the coil using silver epoxy. The array had a Q_ratio_ < 2, which was 33% lower than a comparison PCB‐based coil. In 2021, Kahraman‐Agir et al. demonstrated a wearable coil on an elastane fabric.^104^ Although this was not the first implementation of a wearable coil on an elastane fabric, the effect of phantom variation on coil filling factor was demonstrated. The coil was developed using a silver‐coated polyamide thread with a needle rib technique with an overlay of elastic fabric. The linear DC resistance of the thread was 30 Ω/m, which required the threads to be bundled to lower the effective resistance. Three coil sizes were developed to fit small, medium, and large breast phantoms filled with corn oil. Phantom studies revealed that the SNR of the coil was lower than traditional copper coils. The importance of using a conductive thread with very low DC resistance was apparent, as the low Q_ratio_ and SNR of the fabric coils in both studies were a result of the high conductor resistance.

## 4 STRETCHABLE COILS

Recent receive array developments have added stretchability to flexible coils. A stretchable coil's elasticity enables it to acquire static and kinematic images of the target anatomy across a greater range of the population than an inelastic wearable coil. In cases in which arrays are developed using extremely elastic substrates, the number of individual coil elements required to cover a ROI can be reduced due to the elasticity of the coil when compared with an inelastic wearable coil. This emerging technique involves the development of elastane coils on fabric or, alternatively, integrating nonfabric stretchable coil materials into fabric‐based substrates.

### 4.1 Stretchable coils on fabric

**FIGURE 8:**
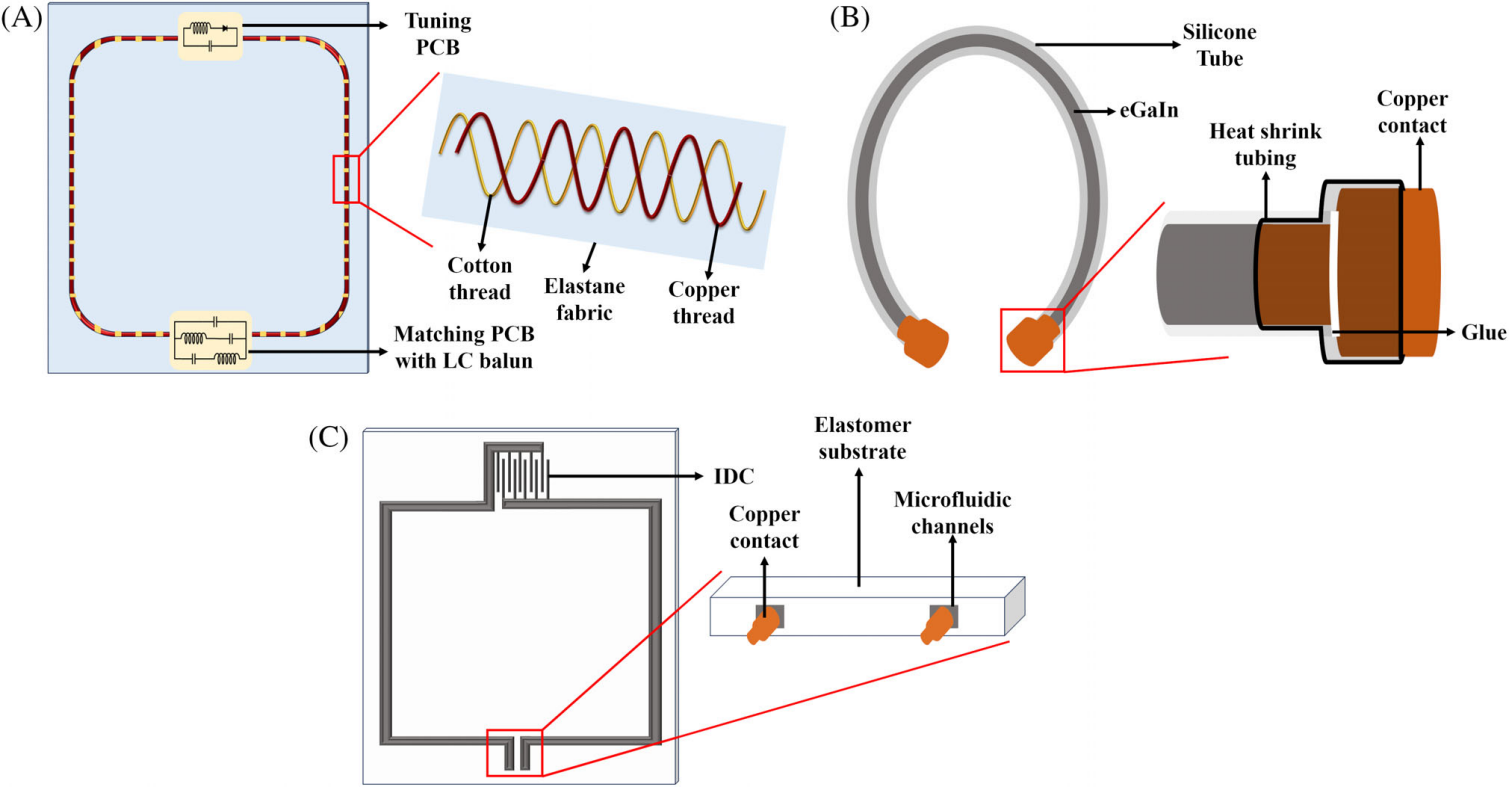
(A) The conductive thread on fabric technique in which copper‐based conductive threads are embroidered on the fabric using normal threads. (B) Stretchable coil developed using liquid metal in a silicone tube. The copper contacts are used to connect the coil to the external circuit, which are pushed into the silicone tube to remove any air cavities at the junction. Glue is then applied at the interface followed by the application of heat shrink tubing for a permanent connection. (C) A stretchable coil with interdigital capacitor (IDC) developed using microfluidic channels containing liquid metal on an elastomer substrate. The coil setup uses copper contacts or copper wires to interface with external circuits.

In 2012, Nordmeyer‐Massner et al. developed the first stretchable coil to image the knee at various flexion angles at 3 T.^105^ The coil conductor was made of numerous thin conductive threads that were formed into a braid of 5‐mm thickness that was sewn onto an elastic fabric. Two rings containing four coils each were mounted onto an elastic fabric using hook‐and‐loop fasteners to form the eight‐channel knee coil. SNR varied across the entire 20% of stretch due to coil impedance variation affecting the preamplifier noise figure; this was mitigated when the coil was noise‐matched at every 5% of stretch. Thus, this work highlighted the need for iterative tuning and matching to maximize SNR in a stretchable coil. Rapid prototyping of stretchable fabric coils using an embroidery machine was introduced for joint and breast imaging at 3 T.^106^, ^107^ The coils were developed by sewing a silver coated conductive thread onto an elastane fabric as shown in Figure 8A. The Q_ratio_ of the unstretched coil equaled that of the PCB coil spaced 4.2 cm above the phantom, highlighting the high linear resistance of the conductive thread coil. The Q_ratio_ of the fabric coil improved when omnidirectionally stretched but worsened for unidirectional stretch; this was validated by lower SNR from phantom studies. The change in decoupling was evaluated by stretching two overlapped conductive thread coils on a phantom, with low decoupling variation across different stretch conditions. However, some changes were observed, and as demonstrated in Nordmeyer‐Massner et al.,^105^ noise‐matching a stretchable array to a baseline state will help minimize SNR variation of in vivo stretchable arrays.

The conductive thread on fabric technique to fabricate stretchable coils has also been adopted for the development of preclinical receive coils.^108^ The overall SNR of the coil was higher than a flexible PCB coil and a commercial surface loop, but the SNR at the periphery was lower than that of the PCB coil, highlighting the need to develop very low resistance conductive threads. The problem can be alleviated by bundling conductive threads, although both inductance and resistance will decrease. A bundle of threads may require manual stitching of the coil loop, as it could fray under the rapid motion of an embroidery machine.

### 4.2 Non‐fabric stretchable coils

Eutectic gallium‐indium (eGaIn) has been the liquid metal of choice for development of the conductive loops in nonfabric stretchable coils. The low toxicity, vapor pressure, and improved skin depth than copper at clinical MRI frequencies make eGaIn an attractive choice to be used in MRI coils and in other wearable electrical engineering applications.^109^, ^110^, ^111^, ^112^, ^113^ In 2017, Varga et al. demonstrated an eGaIn‐based stretchable coil on a neoprene substrate.^114^ The stencil process was used to develop the coil and involved three fabrication steps: to press a polyethylene terephthalate stencil onto the neoprene base, to apply eGaIn to the neoprene base through the stencil, and to remove excess liquid metal by pressing the stencil and swiping a glass slide. Although the Q_ratio_ of the eGaIn coil was about 50% lower than a conventional PCB coil, the neoprene coil managed to achieve a similar SNR distribution due to the proximity of the coil to the ROI. The amount of eGaIn deposited onto the substrate played a key role in determining the strain‐resistance sensitivity of the coil. In the study, a thin layer (˜40 μm in cell size) of eGaIn was well adsorbed on the neoprene base with a cell size ranging between 60 and 100 μm and showed only 5% change in sheet resistance for a 100% stretch. In contrast, excess (non‐adsorbed) eGaIn traces demonstrated a 300% change in resistance for a 100% stretch, which highlighted the importance of trace thickness in this method.

The effect of bending and stretching of the liquid metal coil was studied further at 3 T.^115^ For an 86 × 70 mm coil, the resonance frequency reduced by 2.5 MHz for 10% strain, due to an increase in inductance of the coil. The increase in thickness of the trace from 30 to 100 μm had only a 0.4‐MHz change in frequency, but the resistance of the coil was no longer strain‐insensitive. When the coil was bent around a 50‐mm sample, the resonance change was 0.5 MHz, as the frequency decrease from stretching was counteracted by the increase from bending. The greatest resonance frequency shift (10 MHz) occurred when the coil was stretched by 40%. This study highlighted the importance of coil stretch characterization to improve imaging performance. When a stretchable coil is stretched or compressed, there is a change in resonance frequency and coil impedance.^116^ The stretching of a coil causes the frequency to decrease due to an increase in inductance, and coil compression causes the frequency to increase. For a 100 × 50 mm conductive thread coil, the resonance frequency shifted by 4.3 MHz with a maximum stretch of 40% and shifted by 8.8 MHz with 40% compression. The coils also exhibited SNR reduction of 24% and 30%, respectively, for a maximum stretch and compression of 40%, when compared with the SNR in the unaltered state. Perhaps more importantly, an effective area change affecting the regions that the coil can image was also reported. The effect of load variation in a stretchable coil was studied by loading a preclinical coil with phantom bottles of varying diameters.^108^ A 6.1‐MHz frequency shift was reported with a reduction in return loss for change in bottle diameters from 5 to 1.5 cm. The reduction in SNR can be attributed to the coil becoming more coil noise–dominated with the reductions in bottle diameter. The active and passive methods to overcome the change in SNR due to coil stretch are discussed in more detail in the upcoming sections. To overcome the electrical and mechanical inefficiency of the neoprene coil, a four‐channel stretchable coil was developed using eGaIn in silicone tubes to image the human knee at 3 T.^117^, ^118^ The silicone tubes were cut to the required coil dimensions, and eGaIn, which is a liquid at room temperature, was injected into the silicone tube as shown in Figure 8B. Liquid metal coils with varying diameters of silicone tubing were used to acquire images of a phantom and demonstrated that the liquid metal coil achieved 91% of the copper reference coil SNR. The stretching of the silicone tube alters the contact resistance of the copper insert as a function of the change in tube geometry, which changes the coil sensitivity.

Microfluidic channels in elastomers can be filled with liquid metal to form the conductive loop. This method was used to develop a microfluidic coil at 3 T with an interdigital capacitor (IDC) for automatic passive tuning of the coil when stretched.^119^ The substrate of the coil was developed with a biocompatible elastomer (EcoFlex; Smooth‐On Inc., Macungie, PA, USA). The development of elastomer‐based coils involved creating the top and bottom molds with IDC traces, which was injected with liquid metals such as eGaIn, as shown in Figure 8C. The theory behind the implementation of IDC is to control the frequency shift of the coil when stretched. Coil stretching causes a nonlinear increase of inductance, which reduces the frequency. This can be counteracted by the addition of an IDC whose capacitance decreases as the coil is stretched, thus nullifying the resonance frequency shift. However, in practice, the linear increase in capacitance cannot keep up with the nonlinear increase in inductance resulting in residual frequency shift 10 times smaller than that of a stretchable coil with a fixed capacitor, as demonstrated in this study.^119^ The microfluidic technique was extended to a two‐channel array for knee imaging at 3 T.^120^ Three coil fabrication methods were tested: single layer casting, double layer casting, and direct ink writing. Single layer casting used a negative mold to form overlapping channels and silicone tubing with liquid metal as jumpers but was mechanically unstable. Double layer casting stacked two separate microfluidic coils but resulted in a thick profile. Direct ink writing used spin coating^121^ and 3D printing to produce thin, mechanically, and electrically stable coils. Liquid metal coils suffer from SNR loss when stretched, which can be mitigated using auto‐tuning techniques combined with IDC. EcoFlex is a proton‐rich material visible in MR images and the visibility can be reduced by doping with gadolinium or barium titanate.^122^

## 5 AUTOMATIC TUNING OF STRETCHABLE COILS

Tuning and impedance matching systems are well‐established for antennas and are being widely researched for antenna‐enabled sensors and systems.^123^, ^124^, ^125^, ^126^ Traditionally, resonance frequency shift has not been a concern for MRI, as the rigid conductor maintains its inductance. However, small rigid coils developed using monolithic designs, such as multiturn transmission line resonator technology to observe the NMR spectra at a microscopic scale, require fine adjustment of resonance frequency.^127^ New advances in stretchable and flexible coils have made the frequency shift the forefront of research in MRI. Automatic tuning and matching systems are divided into two subcomponents: resonance frequency detection and resonance frequency adjustment. Two major categories of adjustment systems, which will be discussed, are electronic mechanisms and passive mechanisms.

### 5.1 Electronic tuning and matching

The earliest implementations of automatic tuning and matching systems involved actuators that would drive mechanical trimmer capacitors, controlled by the spectrometer software under a feedback routine.^128^ As coil designs steered away from mechanical trimmer capacitors due to size constraints and availability of smaller capacitors, researchers began exploring other means of automatic tuning including electrically controlled variable capacitors (varactors).^129^, ^130^, ^131^, ^132^ As the reverse voltage bias across a varactor increases, the capacitance also increases. Other investigations used PIN diodes^133^ or micro‐electromechanical system switches^134^ to electrically control a capacitor array.

Many implementations of frequency detection involve generating an excitation signal and detecting voltage or power of the reflected signal. Gulsen et al. and Muftuler et al. directly monitored the resonance frequency using a vector network analyzer.^132^, ^135^ Coils in‐bore were cabled to an externally located network analyzer connected to a computer system. The computer controlled the coil's capacitance based on magnitude and phase measurements from the network analyzer. Venook et al. presented a detection mechanism that placed the coil in series with a reference capacitor.^136^ A frequency sweep was applied to the coil, and the phase difference between the coil and the capacitor was measured. At coil resonance (i.e., no reactive component), the coil was 90° out of phase from the reference capacitor. Although this method takes less than 1 s and performs well for coils with high‐quality factors, it may produce false phase detection if the system senses the zero phase‐crossing above or below the true resonance frequency.^136^

Pavan et al. used an impedance sensing circuit that measures the magnitude and phase of the reflected signal extracted with a directional coupler.^137^ Although this method is accurate for many coil topologies, the tuning and matching process takes up to 4 min to complete. Sohn et al. implemented a system based on a peak power detector, which measured the relative power from the directional coupler.^133^ Their implementation reduced the time to 550 ms per channel. The ArduiTaM system works in a similar manner and can be adapted for field strengths between 1 T and 23 T. Although frequency detection systems that use directional couplers are accurate, their use inside the scanner is often impractical. A microstrip directional coupler requires a length of one quarter wavelength, which for a 3T system is about 58 cm. Miniature directional couplers use ferromagnetic cores, which cannot be used in the scanner. Mehmann et al. used an integrated complementary metal‐oxide‐semiconductor in‐field receiver board to measure the resonance frequency.^138^, ^139^ The receiver board generated an excitation current to the coil and a field programmable gate array–based controller board was used to detect the reflected voltage signal to obtain the coil's frequency response. This was fed into a proportional–integral–derivative controller, which biased series varactors for retuning.

### 5.2 Passive tuning and matching

Passive self‐tuning systems are designed to automatically tune and match coils without an external input. The most common method involves incorporating interdigital capacitors, which change shape as the coil is stretched. Because the capacitance of an interdigital capacitor depends on its dimensions, a carefully designed interdigital capacitor can mitigate the frequency shift due to changes in coil geometry. Motovilova et al. developed self‐tuning stretchable liquid metal coils (eGaIn) with interdigital capacitors in two directions, allowing for bidirectional frequency corrections.^119^, ^120^, ^140^ This work shows a degree of frequency stability at the Larmor frequency for up to 30% stretch. Although mechanical self‐tuning mechanisms show robustness under stretch, additional work is required to evaluate effects if the coil is compressed. The various tuning and matching techniques used in stretchable coils are summarized in Table 1.

**TABLE 1:** Summary of the different implementations of electronic tuning and matching systems.

Reference	Frequency shift mechanism	Frequency detection mechanism
128	Motorized mechanical trimmer	Coupler; envelope detection
132	Varactor	External network analyzer
133	PIN diode switch, capacitor array	Coupler; RF power Detection
134	MEMS switch, capacitor array	Coupler; power detector
135	Varactor	Coupler; external network analyzer
136	Varactor	Reference capacitor; phase comparison
137	Varactor	Coupler; gain and phase detection
138	Varactor	Direct voltage measurement

Abbreviations: MEMS, micro‐electromechanical system; PIN, positive‐intrinsic‐negative; RF, radiofrequency.

## 6 DISCUSSION AND CONCLUSIONS

Wearable and stretchable coils in MRI can adapt to patient anatomy and potentially improve image quality and patient comfort. The various fabrication techniques used to develop flexible, wearable, and stretchable coils are summarized in Table 2. The use of novel materials in the development of coils has also been extended for hybrid imaging modalities such as MR‐guided focused ultrasound.^141^ One notable limitation is that most novel materials and methods used to develop wearable and stretchable coils have not been applied for RF transmission. A primary figure of merit for transmit coils is B_1_^+^ homogeneity while maintaining safe SAR levels, and neither of these benchmarks benefit from closer proximity to the body. Furthermore, the low breakdown voltage and current handling capacity of materials used in wearable technology are unsuitable for high‐power transmission. From a materials perspective, graphene conductors may aid in the adoption of stretchable technology for transmit RF coils. Graphene is a one‐atom‐thick carbon structure in a honeycomb lattice, which provides ballistic motion of electrons. The high tensile strength, fracture strains, and current handling capacity depending on the manufacturing method could make graphene an attractive choice for developing MRI transmit coils.^142^, ^143^ Graphene can be stretched up to 20% of initial length and can be transferred to stretchable substrates.^144^, ^145^ The drawback of using a nascent material such as graphene is the increase in coil cost. The methods to develop and integrate graphene into everyday electronics are still at an early stage, but widespread adoption along with the implementation of new fabrication methods will help lower the cost of graphene‐based coils. The development of transmit coils comes with challenges, such as SAR estimation and variation in power reflection, that is not encountered when developing receive‐only coils.

**TABLE 2:** Summary of the target anatomy, field strength, fabrication methods, and the associated advantages and limitations of various coil development techniques.

Classification	Reference(s)	Target anatomy	Field strength (T)^a^	Coil type: Tx, Rx, Tx/Rx	Fabrication	Advantage(s)	Limitation(s)
Adaptable coils	29	Brain	7	Tx/Rx	Extended ground planes, capacitor patches, knobs for coil adjustment	Easy adjustment of the coil	Manual tuning and matching done for each adjustment
	30	Wrist	3	Rx	Acrylic frame housing connected to a lever for adaptability	Double hump preamplifier matching for improved decoupling	Mechanical coil adjustment
	31	Knee	1.5	Rx	Stretchable conductive braids with copper tape on adjustable coil housing	Optimum SNR for S, M, and L knee sizes	Increased coil fabrication cost and no radial adjustment
	43	Brain	3	Rx	Air pressure induced in bellows connected to coil housing allows coil adjustment	Individual channel adjustment for enhanced interelement decoupling	Increased coil setup complexity for increased channel count
	44	Abdomen and spine	3	Rx	Hinges on the anterior coil housing enable flexibility	Enhanced SNR for pediatric imaging	Semiflexible anterior section with rigid housing
	48	Knee	3	Rx	Modular 8‐channel array with custom interface box	Rearrangement of coils to fit over various anatomies	Limited parallel‐imaging performance
	49	Cardiac	7	Tx/Rx	32‐channel array divided into 8 groups of 4 × 4 arrays	Increased modularity with improved parallel imaging performance	The posterior array arrangement can be uncomfortable for the patient for prolonged scans
	52	Brain	3	Rx	7‐channel modular coil with stacked ground planes	Improved array decoupling	Cumbersome architecture that limits modularity
	53	Neck, ankle, spine, and hip	3	Rx	HIC inspired modular array	Scalable design to cover a wide range of anatomies	Sequences optimization for improved coil performance
	54	Phantom only	9.4 – preclinical	Rx	Ink‐jet printing of coil on a flexible substrate	Rapid, scalable prototyping of flexible coils	Expensive process with precise manufacturing controls
	58, 59, 61, 62	Brain, spine, and lungs	3	Rx	Flexible coils developed using link resonator structure	High conformance with good SNR over a broad range of anatomies	Proprietary manufacturing process
	64	Neck	3	Rx	Spray coating of the coil loop	Coils can be customized to any anatomy	Fine‐tuning of capacitance requires scraping of spray‐coated layers
	68	Brain	3	Rx	Coils developed using vacuum forming and electroless plating methods	Coils can be customized to any anatomy	Expensive manufacturing process
Wearable coils	69	Knee	3	Rx	Flat cable used as a monopole antenna for knee imaging	Large FOV coverage in the longitudinal direction	Difficulty in adapting anatomies other than the knee
	71, 72	Knee and Spine	3	Rx	Screen printing used for developing receive coils	Easy integration into textiles and increased patient comfort for pediatric scans	Fabrication of tuning capacitors can be a tedious process, which requires precise control.
	73	Knee	3	Tx/Rx	Trellis structure–inspired assembly of coil arrays	Modular and wearable array to fit cylindrical geometry	Coil is rigid and inflexible; thus, optimizing coil layout for irregular geometries can be difficult
	77, 81, 82	Neck and Knee	7 and 0.55	Tx/Rx	Coaxial cables used as transmit and receive coils for high and low field MRI	Easy fabrication and scalability for different field strengths	Low Q_ratio_ compared with PCB coils Hot spots were observed at low field strength.
	85, 86, 87, 88	Wrist, breast, and neck	3	Rx	Optimized high‐impedance cables with low impedance preamplifiers to develop MRI coils	Broadband decoupling and current suppression along with enhanced SNR than traditional coils	Coil development at different field strengths require varying core and shield diameters along with different dielectric materials
	89, 90, 91, 92	Head and phantom	3 and 7	Rx and Tx/Rx	Twisted pair cable as Rx only at 3 T and Tx elements at 7 T	Elimination of lumped elements on coil conductor for Rx‐only coils and deformation‐independent decoupling	Insensitive to phantom loading at 7 T
	18, 94, 95	Phantom	7	Tx/Rx	Gapped conductors on either side of a dielectric substrate to form a distributed component model	Low‐profile coil design with no lumped elements for tuning and matching	Restricted 3D deformation due to parallel plate design and limited freedom in decoupling multiple TLR elements
	98, 103, 104	Brachial plexus, neck and phantom	1.5, 3	Rx	Stitching conductive threads or tinned copper braids onto fabric	Rapid coil manufacturing to produce wearable coils	Coil Q_ratio_ and SNR was poor compared to PCB coils
Stretchable coils	105	Knee	3	Rx	Receive arrays developed using conductive braids on fabric	SNR comparable to PCB‐based coils	Conductive threads used to stitch the braids had high resistance and limited coil stretch
	106, 107, 108, 116	Wrist, breast, knee, Preclinical – brain and spine	3, 7 – preclinical	Rx	Embroidering conductive threads onto a fabric	Low‐resistance conductive threads for rapid fabrication of on textile receive arrays	Specialized conductive threads were used and reduced thread tension after multiple stretches
	114	Phantom only	3	Rx	Stencil printing of eGaIn on neoprene	Low‐profile stretchable coil	Precise control over the amount of eGaIn is needed, as coil resistance can vary significantly if excess eGaIn is stenciled
	117, 118	Knee	3	Rx	Liquid metal in silicone tubes integrated into stretchable textiles	Easy manufacturing process to develop stretchable coils for high‐resolution imaging	Contact resistance of copper insert can alter coil sensitivity during stretch
	119, 120	Knee	3	Rx	Microfluidic channels in EcoFlex substrate containing liquid metal	IDC enables passive tuning of the coil, thereby minimizing SNR loss during stretch	EcoFlex is MRI visible

Abbreviations: 3D, three‐dimensional; eGaIn, eutectic gallium‐indium; FOV, field of view; IDC, interdigital capacitor; L, large; M, medium; PCB, printed circuit board; RF, radiofrequency; Rx, receive; S, small; SAR, specific absorption rate; SNR, signal‐to‐noise ratio; TLR, transmission line resonator; Tx, transmit; Tx/Rx, transceive.

^a^ The field strengths not indicated to be preclinical are assumed to be human scanner field strengths.

Flexible PCB coils are packaged within a robust rigid former tailored to the anatomy that houses additional components such as preamplifiers and baluns, thus enabling long‐term use of the coils. However, the inclusion of rigid formers with additional components makes the coil package bulky and compromises ease of handling for the operator. The rigid formers also limit the coil from being near the anatomy to necessitate increased signal reception from the target anatomy. Wearable coils take advantage of the closeness to the anatomy to improve the SNR of the image. However, these coils are developed using materials that are more lossy when compared with copper‐clad PCB coils. The resistive losses of wearable coils prevent them from achieving the theoretical SNR increase. Future research can be aimed toward identifying materials or methods to develop coils that have resistive loss comparable to PCB coils. Graphene is one such example, and the application of copper foil on neoprene fabric can also be explored. Although ease of handling for the operator is improved due to the lightweight wearable coils, such a metric is not easily quantified, as the addition of on‐coil preamplifiers, cable traps, baluns, and cables add bulk and can compromise coil conformity. Because most wearable coils are still prototypes, the long‐term clinical utility of these devices is not available. The improvement in patient comfort is another potential advantage of using wearable coils but difficult to quantify as patient comfort can be subjective.

The phantom and in vivo evaluation of wearable coils should be performed both on curved and flat surfaces. Wearable coils have the advantage over a curved surface due to the omnidirectional conformance of a wearable coil as opposed to the bidirectional or non‐conformance of flexible and rigid PCB coils, respectively. The comparison of SNR over a flat or less curved surface will reveal that the flexible or rigid coil will have the same or better SNR than a wearable coil, but this does not decrease the utility of a wearable coil. The choice of whether a wearable coil or PCB based coil should be used depends on the type of anatomy being imaged. For example, in the case of head or spine imaging, a flexible PCB‐based array in a rigid head former may suffice. However, when musculoskeletal imaging or breast imaging is to be performed, then a wearable coil can be used due to curvature of the anatomical surfaces. For pediatric patients, the type of coil technology to be used depends on whether the MRI is for neonates, infants, children, or adolescents. The sole advantage of using a stretchable coil is the possible reduction in number of elements to cover the same surface area enclosed by a wearable coil. However, this depends on the elasticity of the stretchable substrate and cannot be easily justified if a nonstretchable, wearable coil with sufficient coil elements can cover the same ROI. The major drawback of using a stretchable receive coils is the additional need to implement auto tuning and matching techniques during coil stretch. This can be avoided with a robust preamplifier decoupling in the case of nonstretchable, wearable receive coils to provide comparable SNR across a wide range of the population.

RF coil cabling is a key challenge that needs to be addressed to enable widespread adoption and novel application of wearable coils. Cables make a wearable coil setup bulky and add mechanical stress on coil loops. Conductor failures are aggravated at the edge of the PCB, thus necessitating strain relief of the cable connection. This issue can be mitigated to a certain extent in cases in which the coil conductor can be directly soldered onto the PCB at a cost of increased stress on the coil loop when stretched. In stretchable coils developed using conductive braids, elastomers, and silicone tubes, which use copper foil pads or copper contact inserts for connection to external PCBs, there is a risk of damage to the contacts because of cable twist and movement. High‐density arrays suffer from intercable coupling issues, which pose a threat to patient safety. An MR system coil cable consists of additional components such as baluns and cable traps to suppress common‐mode currents, adding rigidity. Flexible, lightweight, 3D‐printed cable traps have been developed,^146^, ^147^ but scalability remains a challenge. One solution is the use of fiber optic cables for high‐speed data transmission.^148^, ^149^, ^150^, ^151^, ^152^, ^153^ The analog MRI signal is converted into an optical signal at the point of reception and transmitted to the console. Fiber optic cables are light weight and provide high‐speed data transmission, but the loss of data from a damaged fiber optic cable can exceed the analog coaxial counterpart.

Wireless MRI may present an ideal solution to eliminate the use of MR system coil cables. Wireless MRI is a complex technology to implement; only building blocks are available without any commercial wireless RF coils. The high‐power consumption of RF coils will need to be addressed before implementing wireless MRI. Detuning a single‐channel receive coil requires at least 100 mA to achieve the required blocking impedance and scales with increasing channel count. Field‐effect transistor (FET) and gallium nitride (GaN) high electron mobility–powered switches provide alternatives to traditional PIN diode–detuning techniques and can yield current savings with required detuning levels.^154^ GaN FET switches allow unplugged, safe operation of a coil in‐bore without the need for additional passive diode detuning configurations. This will be particularly useful in wireless coils that essentially operate in an unplugged state, thus making GaN FET switches both low‐powered and detuning default safe when compared with traditional PIN diode detuning. Wireless transfer of MRI data has been demonstrated using both analog and digital schemes. The analog scheme uses inductively coupled transmission to send the analog signal from the coil to receivers in the bore^155^, ^156^ and suffers from a reduction in SNR due to inherent analog transmission losses. Digital wireless MRI transmission can also be accomplished with existing techniques using a 2.4‐GHz or 5‐GHz communication link (802.11b or 802.11ad Wi‐Fi standards that specify interfaces for over‐the‐air signaling between two or more devices) and mm‐Wave technology^157^, ^158^, ^159^, ^160^, ^161^ with transmission rates up to 100 Mbps. An mm‐wave‐based Wi‐Gig transmission with transmission speeds of 2.5 Gbps has been demonstrated. Another potential solution may be adoption of the multiple input multiple output technique from 5G technology^162^, ^163^ (cellular technology for local and wide‐area coverage) that can provide transmission speeds from 1 to 9 Gbps; these rates could enable data transmission of any MRI sequence with high‐density coil arrays. There is also ongoing research on wireless power transfer to supply power to on‐coil circuits.^164^, ^165^ MRI coils powered by batteries are possible with rapid innovation in battery technology.^166^, ^167^, ^168^, ^169^ Wireless coils will also need a local reference clock that is synchronized with the scanner.^170^, ^171^, ^172^, ^173^ Extensive reviews of wireless coils are available in literature for an in‐depth understanding of this potentially transformative RF coil technology.^174^, ^175^

Housing of the array elements plays a key role in ensuring patient safety during an MRI scan. The demonstration of safe and effective use of the device is important to obtain regulatory certification for medical devices. The design and development of housing for coils should also be included in the literature. Adaptable coils house the coil elements in a rigid yet adjustable former for MRI, thus ensuring safe coil operation. However, there is a lack of housing design for wearable and stretchable coils in literature. The wearable coils are near the anatomy and need to be housed appropriately to prevent excessive SAR and tissue heating. This is very important in the case of transceive coils such as coaxial cable coils, TLR coils, and twisted pair coils. The FACE coil^87^ incorporates HIC elements in a thermoplastic polyurethane housing with an opening at the front to prevent patient claustrophobia. The bra coil^86^ houses four elements between two cushion layers enclosed by medical grade synthetic leather. Further research is needed to house on‐fabric wearable coils without compromising the conformity of the coil. The development of small form capacitors and inductors that can be directly incorporated on the wearable coil loop is another area of potential research. Current wearable coil development methods use PCBs with lumped elements incorporated in the coil loop using copper contacts or copper foil as bridges that can compromise coil durability, conformance, and mechanical stability. Efforts are needed to implement robust, rapid, low‐cost, large‐scale manufacturing of wearable coils. There is also research potential to address the durability and electrical reliability of a wearable coil before achieving the goal of combining wireless and wearable MRI technologies. The integration of wireless technology into wearable coils presents an ideal solution for improved patient experience in an MRI scanner and other MRI‐based hybrid imaging modalities, but the roadmap to achieving that goal is through the integration of material and electrical engineering techniques to overcome the limitations of current MRI hardware to present accessible and affordable MRI scans to patients.
